# Toward a New Approach to Investigate the Role of Working Memory in Stereotype Threat Effects

**DOI:** 10.3390/brainsci12121647

**Published:** 2022-12-01

**Authors:** Margaux Piroelle, Marlène Abadie, Isabelle Régner

**Affiliations:** Laboratoire de Psychologie Cognitive, Aix Marseille Université, CNRS, 13331 Marseille, France

**Keywords:** stereotype threat, aging, working memory, regulatory fit

## Abstract

Stereotype threat arises when the activation of negative stereotypes about a group impairs performance of stigmatized individuals on stereotype relevant tasks. There is ample evidence that stereotype threat leads to performance detriments by consuming executive resources. Several studies indeed showed that working memory (WM) mediates stereotype threat effects among young adults. More recently, researchers have sought to understand whether the same mechanisms underlie age-based stereotype threat, but findings are mixed regarding the role of WM and some authors rather favor a motivational explanation based on regulatory fit. The present review critically appraises the empirical support for distinct forms of stereotype threat effects mediated by distinct mechanisms. We propose a novel approach based on one of the most recent WM models, the time-based resource sharing model, to evaluate the impact of stereotype threat on attentional resources in WM among both young and older adults.

## 1. Introduction

Many groups are targeted by negative stereotypes, for example, girls and women in science [1], ethnic minorities in intellectual abilities [2], and older adults in memory [3]. Research on stereotype threat demonstrates that the context of evaluation influences task achievement for negatively stereotyped individuals. Performing a task in a context in which there might be a risk of confirming negative stereotypes about one’s group can lead to underperformance [4,5]. It was first demonstrated by Steele and Aronson [6] with Afro-American students on standardized intellectual test performance. Since then, many researchers have sought to understand how this effect occurs. One of the most prevalent explanations of stereotype threat effects on performance is the integrated process model [7]. According to this model, stereotype threat effects involve cognitive and emotional processes that draw on executive resources, particularly on working memory (WM) resources. The reduction in these resources results in task underperformance because the few resources left are not sufficient to handle the task correctly.

WM is a key component of human cognition, conceived as a system that uses a capacity-limited resource, most often conceptualized as attention, to temporally maintain and process a small amount of information during ongoing cognition [8,9,10,11]. The role of WM in stereotype threat effects has been extensively studied in young adults. Using typical WM tasks, such as the complex span task [12,13], several studies have demonstrated that stereotype threat impairs WM performance in young adults [14,15,16,17]. The role of WM in age-based stereotype threat has also been investigated, but results are mixed and do not allow one to state on its involvement in this phenomenon [18,19,20]. These contradictory findings have led to alternative assumptions that are still debated in the literature [21,22].

The purpose of this article is twofold. First, it aims to critically review the evidence for the role of WM in stereotype threat effects. Specifically, we show that measures of WM used in stereotype threat studies are not completely reliable, which might have led to an underestimation of the effects of stereotype threat on WM and explains the mixed findings regarding these effects in older adults. Second, we propose a novel and original approach to investigate the role of WM inspired by recent WM models [9], which provide fine-grained techniques to assess the amount of resources involved in a task. Developing a better measure to assess attention is the first step toward better understanding the mechanisms underlying stereotype threat effects. In the following, we first review the studies on young adults, then examine the mixed findings of studies on older adults, then describe the two main explanatory theories of stereotype threat effects, and finally develop our new approach. 

## 2. Stereotype Threat and Working Memory in Young Adults

The construct of WM has regularly been used to explain effects in the stereotype threat literature since its inception [16,23]. Since then, several studies have provided evidence supporting this assumption and the role of WM has been examined from different perspectives, which we review below. 

### 2.1. Working Memory as a Mediator of Stereotype Threat Effects 

Schmader and Johns [16] provided initial evidence that stereotype threat reduces WM task performance. In two experiments, they assessed the effect of gender-based (Experiment 1) and ethnic minority-based (Experiment 2) stereotype threats on recall performance in a typical WM task, the operation span task (OSPAN) [24]. Participants were asked to memorize verbal information (e.g., words) while evaluating, between the presentation of each item to be remembered, the accuracy of mathematical equations (processing task). Sets of three to five words were presented and WM capacity was assessed as the total number of words correctly recalled from perfectly recalled sets (i.e., absolute span score). Negatively stereotyped groups (women in Experiment 1 and Latinas in Experiment 2) were compared to positively stereotyped participants (e.g., individuals for whom these stereotypes are favorable; men in Experiment 1 and Whites in Experiment 2). The context in which the task was performed was typically manipulated in order to induce or to reduce stereotype threat. In the stereotype threat condition, the task instructions emphasize the evaluation of the negatively stereotyped domain (e.g., the task was described as related to mathematical ability in Experiment 1 or as related to performance on intelligence tests in Experiment 2). On the contrary, in the reduced threat condition, concerns about taking a test in a domain in which one’s group is negatively stereotyped is minimized (e.g., the task was described as assessing WM). Results showed that the negatively stereotyped groups recalled fewer words in the stereotype threat condition than in the reduced threat condition and compared to the positively stereotyped groups in the stereotype threat condition only. 

In a third experiment, Schmader and Johns [16] examined the role of WM in mediating the effect of stereotype threat on performance in an alternative math task. A sample of women, under the stereotype threat or reduced threat condition, first performed a WM task not described as related to mathematical abilities, followed by a math test. The WM task was similar to the OSPAN task, except that they were asked to count the number of vowels in sentences instead of verifying equations. Results showed that women under stereotype threat recalled fewer words in the WM task and were less accurate in the math task than those in the reduced threat condition. Most importantly, WM capacity mediated the effect of stereotype threat on math performance, which means that the reduction in math performance in the stereotype threat condition was caused by the decrease in WM capacity. 

Following this, other studies tested the mediating role of WM. Johns et al. [14] investigated the role of emotion regulation in gender-based stereotype threat effect. In the stereotype threat condition, women were evaluated by men and informed that the task was a math test, whereas in the reduced threat condition, they were evaluated by women and the task was described as problem-solving. WM capacity was then evaluated by using the absolute span score in a reading span task, where words to be remembered (from four to six) were interspersed by sentences in which the number of vowels had to be counted. This task was described as a part of an unrelated study. After the WM task, participants performed a math test. Results showed that decrease in math performance under stereotype threat might be due to the regulation of anxiety by suppression processes of negative thoughts and emotions, which disrupts WM efficiency (Experiment 3). Moreover, inducing a most efficient regulation strategy alleviated the impact of stereotype threat on WM, thus not reducing performance on the subsequent math task. This shows that spontaneous emotion regulation processes when facing stereotype threat can draw resources from WM. 

Rydell et al. [15] found that WM capacity (captured from the total number of words correctly recalled in a reading span task) mediated the effect of the activation of different types of identities on math performance (Experiment 3). The WM task was described as unrelated to math ability. When women’s gender identity was made salient (by being informed that the purpose of the study was to investigate why men are better than women at math), stereotype threat decreased their math performance by reducing their WM capacity. In contrast, when a positive identity was activated (by being informed that the purpose of the study was to investigate why college students are better than non-college students at math), the effect of stereotype threat on math performance was counteracted because WM resources were no longer depleted. 

Other research investigated the impact of feelings of power on stereotype threat effects on women’s math performance and its impact on WM [17]. Stereotype threat was activated by indicating that the purpose of the study was to investigate why women are worse than men in math (stereotype threat condition) while in the reduced threat condition, no mention to gender was made. The WM task, presented as visual processing, was a letter memory task, in which sequences of letters (from five to nine) were displayed sequentially. Participants had to continuously keep in mind the last three letters displayed and recall these three letters when prompted (at the end of the sequence). Women’s WM capacity (i.e., the proportion of letters triads correctly recalled) and math performance were not affected by stereotype threat when a high feeling of power was induced. However, in the low or no feeling of power conditions, a stereotype-threat pattern emerged, mediated by WM capacity (Experiment 3). 

Forbes and Schmader [25] found a different, but not opposite, pattern on the mediating role of WM. They investigated the impact of gender stereotypes and attitudes toward math on a reading span task and a math task in a context of stereotype threat. As in previous studies, the WM task was not described as related to math. The women completed the study, which was either described as diagnostic of their mathematical abilities and had to indicate their gender (stereotype threat) or as a problem-solving task (reduced threat). Training participants to have more positive attitudes toward math (i.e., reinforcing “I like math”) had no effect on WM and math performance. In contrast, women trained to associate their gender with being good at math exhibited increased WM and math performance in the stereotype threat condition than in the reduced threat condition. WM mediated the effect of stereotype threat on math performance. Training women to associate their gender with being good at math might have a reduced negative affect and cognitive load, which improves their WM and math performance.

Although few in number, these studies clearly demonstrate that stereotype threat impacts performance in cognitive tasks due to the depletion of WM resources. These studies measured WM capacity as a whole and did not take into account individual differences in WM capacity that might affect the impact of stereotype threat on performance. Other research investigated the moderating role of individuals’ WM capacity in stereotype threat effects on performance. 

### 2.2. Individual Differences in Working Memory Capacity as a Moderator of Stereotype Threat Effects 

In line with the findings that stereotype threat draws on WM resources, it has been hypothesized that high-WM-capacity individuals are better able to cope with stereotype threat [7]. Régner et al. [26] tested this moderation assumption among engineering students. WM capacity was assessed with a reading span task in which participants were asked to remember letters (from two to five) interspersed by sentences to be judged as being meaningful or not. In the stereotype threat condition, participants were simply told that the task was diagnostic of logical reasoning ability, whereas in the reduced stereotype threat condition, the same task was presented as a gender-fair diagnostic of logical reasoning ability. After the stereotype threat condition manipulation, they performed a complex reasoning ability task. Reasoning accuracy of high WM capacity participants (women and men) was not affected by stereotype threat. By contrast, the typical stereotype threat pattern appeared in low-WM-capacity women. Low-WM-capacity women underperformed relative to low-WM-capacity men when they were told that the test was diagnostic and performed as well as men when they were told that the test was diagnostic but gender-fair. This finding was replicated using a similar methodology among secondary school students [27]. 

Hutchison et al. [28] also showed that in men, stereotype threat effects are moderated by individual differences in WM capacity. First, participants performed an OSPAN task to measure their WM capacity, which was indexed by the total number of letters recalled in sets perfectly recalled. Then, stereotype threat was manipulated: in the stereotype threat condition, the task was described as measuring verbal skills in men and women and they indicated their gender, while in the reduced threat condition, it was described as measuring processing skills. Finally, participants performed a Stroop task [29]. Results showed that stereotype threat interfered with goal maintenance on the Stroop task, only for low-WM-capacity individuals. 

Individual differences in WM capacity moderate the stereotype threat effects on performance in cognitive tasks. Even with a reduction in WM resources caused by stereotype threat, high-WM-capacity individuals have enough resources left to perform the task successfully. By contrast, the few WM resources available under stereotype threat for individuals with low WM capacity are not sufficient to succeed at the task. This finding suggests that stereotype threat may actually reduce WM resources, because it impacts individuals with high versus low WM resources differently.

### 2.3. The Impact of Stereotype Threat Effects on Working Memory Task Performance

Other research examined stereotype threat effects on WM performance without investigating it as a mediator or a moderator of the effects of the threat on task performance. In these studies, WM tasks were depicted as measuring stereotyped abilities. Schmader et al. [30] demonstrated that affective and cognitive processes interact under gender-based stereotype threat (Experiment 2). They used a reading span task where participants had to memorize four to six words with a sentence to read in between each word. Sentences were manipulated such that one sentence per set was either confidence- or doubt-related. In the stereotype threat condition, the purpose of the study was presented as investigating gender differences in a diagnostic math test, while in the reduced threat condition, it was presented as investigating individual differences in problem solving. WM performance was impaired by stereotype threat for participants with higher levels of anxiety primed with doubt. By contrast, those primed with confidence did not exhibit a stereotype threat pattern. 

Underperformance in WM tasks under stereotype threat was also found in a study investigating gender-based stereotype threat [31], when examining the impact of stereotype threat among multiple minority identities [32], with ethnic minority children [33], and adults and children with obesity [34,35]. Further evidence comes from studies examining diagnosis threat. Diagnosis threat is experienced by people with a traumatic brain injury, who are stereotyped as less cognitively efficient. When given typical tests of neuropsychological clinical assessment, they performed worse on WM tests under stereotype threat than under reduced threat conditions [36,37,38]. Finally, Beilock et al. [39] investigated the impact of gender-based stereotype threat on domain-specific WM components [40], using simple and complex modular arithmetic problems presented either horizontally or vertically, respectively, which are supposed to rely on phonological or visuo-spatial resources in WM. Stereotype threat impaired performance in complex problems relying on phonological resources in WM only, and it elicited thoughts of worries. 

The reduction in WM performance caused by stereotype threat has been shown in studies in which WM was not examined as a mediator or a moderator. Research using WM tasks to investigate different aspects of stereotype threat, but not in order to examine its impact on WM, also found a deleterious effect of stereotype threat on WM performance. Although these studies showed a negative impact of stereotype threat on WM performance, this may simply reflect the impact that stereotype threat could have on any task rather than a reduction in WM resources per se, as these tasks have been described as measuring the stereotyped domain. 

### 2.4. Summary of Findings and Critical Assessment of WM Measures Used in Young Adult Studies

WM has been a focus of interest to understand how stereotype threat undermines performance in young adults. Evidence reviewed here shows that stereotype threat impairs WM performance [30,31,32,33,34,35,36,37,38,39], its effect on cognitive task performance is higher in low-WM-capacity individuals [26,27,28], and WM recall performance mediates its effect on cognitive performance [14,15,16,17]. According to the integrated process model, this alteration in WM recall performance may reflect a reduction in executive resources [7]. In these studies, classic complex span tasks, such as OSPAN or reading span tasks, are mostly used to assess WM performance. They are, in essence, dual tasks, with both a memory (storage) and a processing component. The processing component (e.g., verifying math operations or judging whether sentences are meaningful or not) is interweaved between the to-be-remembered items (e.g., words or letters) to prevent their maintenance, thus serving as a distractor. Participants should maintain active representations of the to-be-remembered items in the face of distraction. To obtain a reliable measure of WM capacity with these tasks, it is important to ensure that participants are fully attending to the processing portion of the task. However, processing task performance is not often reported when assessing WM in stereotype threat studies. Thus, stereotype threat effect may have been underestimated because it may have impacted both the processing and the memory component of the task, while its effect is almost only measured on the memory component. Furthermore, it is important that the processing portion of the task be adjusted to each individual’s WM capacity to avoid the task from being more attentionally demanding for one participant relative to another [41]. If this adjustment was not made, this could definitely lead to interpretation issues in terms of resources, because stereotype threat effect could appear on the memory performance of an individual for whom the processing task is very demanding, but not for another individual for whom the processing task is undemanding. The problem is exacerbated in studies conducted with elderly individuals, as their performance in cognitive tasks is highly variable. Thus, the effect of stereotype threat on WM resources could not be properly estimated in previous stereotype threat studies since the WM measure itself was unreliable.

## 3. Stereotype Threat and Working Memory in Older Adults

Stereotype threat effects in cognitive performance in older adults have been widely demonstrated, particularly in memory [42,43]. Performance of older adults can be impaired simply by characterizing the task as assessing memory capacity [44,45] and by mentioning the presence of younger adults in the experiment [18,46]. Stereotype threat also disrupts performance in clinical neuropsychological tests used for the assessment of neurodegenerative diseases [47,48,49]. Thus, negative stereotypes about aging can be particularly damaging in the daily life of older adults [50]. These stereotypes are particular in that they can predict cognitive and physical declines, as they are actually true, although there is great variability among individuals in the propensity for age-related cognitive decline [51,52]. Thus, confirming negative stereotypes about aging is a real threat to older adults, particularly with respect to neurodegenerative diseases, which are of great concern to them. By contrast, stereotypes about young adults are false assumptions of inferiorities (e.g., black people are less intelligent than white people). Thus, stereotype threat might be experienced differently in older and younger adults. Because cognitive decline also affects WM efficiency, the mechanisms underlying age-based stereotype threat may not be the same as for younger adults. 

### 3.1. The Role of Working Memory in Age-Based Stereotype Threat: Contradictory Results

The study of Hess et al. [19] was the first to examine the role of WM capacity in age-based stereotype threat effect on long-term memory performance. Older adults (60–82-year-olds) were either informed that the purpose of the study was to explain why younger and older adults perform differently on memory tests and asked to write down their age (stereotype threat condition), or they were informed that the test they were going to take was free of age-related biases (reduced threat condition). A computation span task was used to measure WM capacity, but this task was, however, not described as related to memory. Older adults were asked to solve three to seven simple arithmetic problems (e.g., “5 + 2”) and to memorize the second number of each problem (e.g., “2”). Next, they performed a long-term memory task in which they had 2 min to memorize 30 words belonging to 6 different semantic categories. They were then asked to write down every word they could remember. Results indicated that long-term recall was reduced in the stereotype threat condition and that this effect was most evident for young-old adults (60–70-year-olds). However, unexpectedly, WM performance (total number of correctly recalled items and span score) was not impaired by stereotype threat, even though the computation span occurred sooner after the threat manipulation than the long-term memory task. The authors concluded that stereotype threat effects may depend on the association between the stereotype and the skill as measured by the task. Because age-based stereotype threat affects memory in particular, explicitly presenting the task as an assessment of memory, as was the case with the long-term memory task, would increase its impacts compared to when the task is not explicitly presented as measuring memory, as was the case with the computation task.

In research comparing young and older adults using stereotype-relevant manipulations for each group, the preservation of WM under stereotype threat among older adults has been replicated [20]. The manipulation for young adults involved fabricated stereotypes regarding their college majors, whereas older adults were exposed to age-related stereotypes. In the stereotype threat condition, older adults were told that the study investigated cognitive ability, which younger adults tend to be better at than older adults. In the positive stereotype condition, the study was presented as investigating verbal and analytic reasoning, which older adults tend to be better at than younger adults. WM was captured with the OSPAN task, which was not presented as related to memory. Results showed that stereotype threat lowered WM capacity (i.e., the total number of correctly recalled words) of young adults, but not of older adults. 

Another study examined the differential effect of stereotype threat on encoding and retrieval in long-term memory, while also assessing its effect on WM [53]. Older adults performed a long-term memory task in either a stereotype threat condition, in which the stereotype activation was made either before or after the encoding phase, or in a reduced-threat condition. They read scientific findings about age-related decline (stereotype threat) or about language which is age-neutral. After the long-term memory task, they completed a general knowledge task and a backward digit span task to capture their WM capacity. In this task, strings of digits were displayed orally and had to be repeated in reverse order; the length of the strings increased every two trials until participants failed at two strings of the same length. They were also asked to form mental images of three objects they had heard prior to the backward digit span and to recall their names after the task. Their long-term memory performance was lowered under stereotype threat only when it was activated before encoding. Thus, stereotype threat could alter the efficiency of encoding in memory, which affects subsequent retrieval. No effect of the stereotype manipulation appeared on WM performance (i.e., the number of correctly recalled digit strings, and the number of recalled object names), but the threat effect may have been weakened by performing multiple tasks between stereotype activation and completion of the WM task. 

Contradicting these results, other research provided support for the impact of age-based stereotype threat on WM. Mazerolle et al. [18] conducted a study where young and older adults performed a reading span task, described as diagnostic of memory capacity, in which they had to read two to five sentences and remember the last word of each sentence. This was then followed by a cued-recall task, which is a memory task allowing one to dissociate control and automatic processes in memory using the process-dissociation procedure. Participants were told that both younger and older adults were participating in the study, and these tasks were described as age-neutral in the reduced threat condition. Contrary to previous results, older adults exhibited poorer WM performance in the stereotype threat condition than in the reduced threat condition. They underperformed compared to younger adults in the stereotype threat condition, while there was no difference between these two groups in the reduced threat condition. Stereotype threat also enhanced the use of automatic processes in memory and decreased the use of controlled memory processes. These results provide evidence that age-based stereotype threat might interfere with WM resources. 

Supporting this, Barber and Mather [21] also found a reduction in WM performance (i.e., total number of words correctly recalled on a reading span task), for older adults in a stereotype threat condition compared to a reduced threat condition, depending on the task reward construction. In this study, stereotype threat was activated by reading a fictional article on age-related memory decline followed by asking participants’ age, whereas in the reduced threat condition, the article explained that memory was preserved from age effects. Other studies using different types of WM tasks such as digit span tasks also found reduced WM performance in older adults under stereotype threat (the study was described as assessing why older adults perform more poorly than younger adults on intellectual tests) compared to a reduced threat condition (the study was described as assessing interindividual differences) [54,55]. 

A study found a moderating effect of WM capacity on age-based stereotype threat effect on driving [55]. Older adults carried out a driving task under stereotype threat or in a reduced threat condition. Then, during a second session, which took place from 1 to 14 days later, their WM capacity was assessed with the OSPAN task. Results showed that stereotype threat disrupted the driving efficiency of low-WM-capacity older adults, but not that of those with high WM capacity. This finding is consistent with the moderating role of WM in the stereotype threat effects in young adults [26,27,28]. It may be noted, however, that the WM task instructions were not reported in this study. Therefore, it is possible that the manner in which the WM task was presented, which may have been threatening for older adults, interfered with performance. Other research has found no moderating effect of WM capacity on age-based stereotype threat effects [20,21,46]. 

Finally, a study investigated mind wandering (i.e., off-task thoughts related or not to the task) as a plausible explanation of stereotype threat effects on WM performance. In the stereotype threat condition, younger and older adults read a fictional article about age-related memory decline and were told that they would take a memory test, whereas in the reduced threat condition, the article was about age-related memory changes as a function of lifestyle, and they were told that they would take a math test. Then, they performed an OSPAN task. Older adults under stereotype threat exhibited more mind wandering about the task and poorer recall accuracy than those in the reduced threat condition and younger adults. 

The role of WM in age-based stereotype threat has been examined in several studies that have yielded conflicting results. These could be due to methodological differences. 

### 3.2. Methodological Differences That May Account for Conflicting Results in Older Adult Studies

WM is impaired by age-based stereotype threat only when the task measuring it is described as relevant to the stereotype [18,21,54,56]. By contrast, when the WM assessment is correlational, measured by an additional task that is not depicted as relevant to the stereotype, it remains unaffected by the threat [19,20]. However, in young adults, studies demonstrating that WM capacity mediates the impact of stereotype threat on performance used correlational assessment, and the WM task was not always described as relevant for the stereotype [14,15,16,17]. Thus, findings supporting the impairment of WM under stereotype threat could be due to the fact that any task presented as memory-related could be impaired by stereotype threat in older adults. This effect has been shown in a study describing an arithmetic task (a domain free from aging stereotypes) as related to memory [57]. However, in studies using WM tasks not described as memory-related, the task instructions may have led participants to focus on the processing component of the task. For example, Hess et al. [19] asked participants to perform a WM task described as a quantitative task, in which they had to solve arithmetic equations while remembering the second number of each equation. The description of the task goal could have led participants to focus and prioritize performance of the processing component over the memory component. Therefore, stereotype threat should have impaired performance on the processing component, but only memory performance was reported. Furthermore, the type of activation of stereotype threat must also be considered. It can be induced by different ways: fact-based manipulations explicitly expose the stereotype and task performance expectations (e.g., “Older adults perform worse than younger adults on memory tasks.”) while stereotype-based manipulations use more subtle cues of threat (e.g., “Younger adults also participate in this experiment.”). Two meta-analyses have shown that stereotype-based manipulations alter performance more than fact-based manipulations in older adults [42,43]. The former type of activation would be more ambiguous, create more mind wandering, and be more attentionally demanding, thus impairing WM performance. Failure to find any effect of stereotype threat on WM in older adults might be due to the type of activation used in these studies, which were either fact-based [20,53] or moderately explicit [19]. Importantly, Amstrong et al.’s meta-analysis [43] also demonstrated that the effect size of stereotype threat effect on older adults is larger for WM than for episodic memory tasks. 

Finally, as in young adult studies, traditional dual tasks were used to assess WM performance, but mostly without controlling for the processing component of the task. The use of such an unreliable measure of WM could also explain the conflicting results obtained in older adult studies. First, stereotype threat may have impacted either the processing component or memory component of the task, whereas its effect is measured only on the memory component of the task. In addition, the difficulty of the processing task was not adjusted for individuals’ WM capacity, which is highly problematic because of the high interindividual variability of these tasks in aging. As a result, WM tasks may have been very demanding for some individuals and undemanding for others. If stereotype threat effects draw on attentional resources, they could appear on the memory or processing components of the task, but only when individuals no longer have sufficient resources to complete the task nor manage the negative thoughts generated by the stereotype activation.

## 4. Main Explanatory Theories of Stereotype Threat Effects 

### 4.1. The Integrated Process Model of Stereotype Threat

According to the well-known integrated process model proposed by Schmader et al. [7], stereotype threat effects involve cognitive and emotional processes. Situations eliciting the risk of confirming a negative stereotype about one’s group generate a physiological stress response, increase alertness, and trigger negative thoughts and emotions. These responses to threat lead individuals to engage in emotion regulation in an attempt to suppress these negative affects. Together, these processes tax WM resources, conceptualized as attentional executive resources responsible for maintaining goal representations in the face of interference and distraction [58,59]. In turn, these resources are not available for task completion, thus impairing performance. 

As reviewed above, this model has been well demonstrated in young adults; there is ample empirical evidence that stereotype threat effect impacts WM performance, and even that WM performance mediates the effect of the threat on other cognitive task performance [14,15,16,17]. However, it should be noted that the mediating role of WM on stereotype threat has only been tested a few times. In addition, the mediating role of WM failed to be replicated in age-based stereotype threat studies [19]. These inconclusive results led the researchers to propose another theoretical approach to explain this phenomenon in the elderly. 

### 4.2. The Regulatory Focus Model of Stereotype Threat

Seibt and Förster [60] were the first to draw on the regulatory focus theory to study the effect of stereotype threat on the strategies adopted by young adults when performing cognitive tasks. According to this theory [61], each individual is oriented toward a promotion or prevention goal in their strategic approach to cognitive tasks. Prevention focus goes hand in hand with safety and responsibility, which leads to sensitivity to losses and errors. In contrast, people with a promotion focus are driven by accomplishments and aspirations; they chase gains and success. Individuals’ regulatory focus is stable over time, but it can be changed in the short term by situational factors. Seibt and Förster [60] showed that stereotype threat leads to a prevention focus and a vigilant, risk-averse strategic approach. In the regulatory focus theory, the regulatory focus interacts with the task, facilitating or interfering with performance. When the task goal matches the individual’s orientation (e.g., when a promotion-oriented person performs a task that promotes gains), there is a regulatory fit, and task performance is enhanced. In contrast, if the task goal does not match the individual’s orientation, there is a regulatory misfit, and task performance is reduced [62,63,64,65]. Thus, if stereotype threat leads to a prevention focus, its deleterious effect should appear when the task is gain-oriented (e.g., succeeding at the task), but disappear when the task is loss-avoidance-oriented (e.g., avoiding errors) [66].

Barber and Mather [21] investigated the role of WM and regulatory focus to understand mechanisms underlying age-based stereotype threat. Older adults performed a sentence span WM task under stereotype threat or reduced threat conditions. The task was gain- or loss-oriented. In the loss-oriented condition, participants were given 100 poker chips at the beginning of the experiment and told that they would lose three for each word they forgot. In the gain-oriented condition, they had no poker chips at the beginning of the experiment and gained two chips each time they recalled a correct word. In both conditions, they were rewarded according to the number of poker chips they had at the end of the experiment. Results showed that stereotype threat interacts with task orientation. In the stereotype threat condition, WM performance was impaired when the task was gain-oriented, but the effect disappeared in the loss-oriented condition. Moreover, in the loss-oriented condition, participants in the stereotype threat condition outperformed those in the reduced threat condition. These findings suggest that stereotype threat impairs WM performance only when there is a misfit between the task goal and participants’ orientation. Orienting the task goal toward loss avoidance counteracts the deleterious effect of stereotype threat on performance. This finding was replicated in older adults on memory tasks and in cognitive status assessments [67,68]. Older adults are more conservative in their responses and make fewer errors under stereotype threat [67,69], showing that they favor a prevention-oriented strategic approach. 

Popham and Hess [20] also investigated the regulatory focus account in a study with young and older adults. After the stereotype threat manipulation, individuals’ orientation was assessed with a letter-cancelling task that required them to cross out a sequence of two letters within a large sequence of letters. The strategic approach of the task was characterized by measuring processing speed and accuracy (i.e., a prevention focus should result in reduced speed and better accuracy). On the letter-cancelling task, both age groups exhibited a pattern of prevention; this, however was stronger in older adults. Then, participants performed an OSPAN task. Emotion regulation capacities were assessed with questionnaires a few days prior to the experiment. Results showed that older adults had better emotional control than younger adults. Emotional control moderated stereotype threat effect on WM performance in younger adults only: younger adults with lower emotional control were affected by the threat, whereas those with higher control were not.

For Barber [22], older adults may not experience a reduction in WM resources due to suppression processes engaged during stereotype threat because they are better at regulating their emotions. Thus, the deleterious effect of age-based stereotype threat on performance would be due to the misfit between the prevention orientation and the generally gain-oriented goal of cognitive tasks. In contrast, the two proposed mechanisms—attentional resources and regulatory focus—would be at play in younger adults under stereotype threat: they would be more prevention-oriented and/or would undergo a decrease in executive resources. However, although the effect of regulatory fit in stereotype threat has been shown several times in young and older adults, the underlying cognitive mechanisms are still unknown. In addition, a consensus has yet to be reached among researchers on which of the two proposed models best accounts for the age-based stereotype threat effect. 

### 4.3. Confrontation between the Two Models

Little is known about the mechanisms underlying the regulatory fit or misfit effect on performance. Regulatory fit has been proposed to lead to a “feeling-right experience”, leading to an increase in self-confidence and self-control, which in turn enhances motivation and engagement in the task [65,70,71,72]. However, some studies have shown that stereotype threat may also lead to increased motivation and task engagement in a misfit situation [73,74]. One study examined the role of regulatory focus on task performance with individuals under high- (prevention-oriented) or low-pressure (promotion-oriented) conditions [75]. Results showed that when the task used was high-attention-demanding, participants in the high-pressure condition performed better than those in the low-pressure condition when the task was loss-oriented, but the opposite pattern appeared when the task was gain-oriented. By contrast, this regulatory fit pattern of results was not found, and was even reversed, when a low-attention-demanding classification task was used. The authors argued that a fit between the task (loss- or gain-oriented) and the individual’s regulatory focus (high vs low pressure) increases executive resources in relation to the sense of “feeling-right”. By contrast, a regulatory misfit would draw on executive resources as the absence of the “feeling-right experience” could lead to worry and anxiety. 

Although no study has precisely measured the effect of regulatory fit or misfit on attentional resources, some have shown impaired WM performance under regulatory misfit conditions in both younger and older adults. These studies typically used gain-oriented WM tasks while participants were prevention-oriented by the threat. Thus, it is possible that the inconsistency between individuals’ regulatory focus and the task can draw on attentional resources. 

Other similarities between the two models can be noted. For example, Seibt and Förster [60] showed that under stereotype threat, prevention-oriented young adults had less flexibility and creativity, were more perseverative, and used more analytic thinking than those who were promotion-oriented. Thus, prevention focus could be related to an increased use of control or executive processes. Other studies indicated that stereotype threat alters the selection and execution of strategies and leads to more strategy repetition across trials in older adults [57,76]. Strategy use also mediates the impact of stereotype threat on performance in these studies. These findings can be interpreted in two ways: on the one hand, according to the regulatory focus perspective, the alteration of strategy selection and execution suggests that older adults become prevention-oriented under stereotype threat. As these tasks were gain-oriented, the misfit between the prevention focus and tasks impaired performance. On the other hand, according to the integrated process model, these strategical alterations could be a consequence of executive resource depletion caused by the threat. 

To sum up, the basis of the regulatory focus theory is motivational, but cognitive processes, such as attentional resources, could also contribute to the regulatory fit and misfit effects. However, the impact of stereotype threat and regulatory misfit on attentional resources have never been quantified. Developing a better measure to assess attentional resources is a first step toward a better understanding of the mechanisms underlying stereotype threat effects among both young and older adults. 

## 5. Investigating the Role of Attentional Resources in Working Memory in Stereotype Threat Effects

In early papers about stereotype threat effects, researchers suggested that this phenomenon could interfere with the allocation of attention [6] or decrease cognitive resources by increasing cognitive load [23], and therefore investigated the role of WM [16]. Most of these studies used recall performance or absolute span score in complex span tasks (e.g., OSPAN task), as an indicator of WM capacity. These studies have made major advances in understanding the mechanisms underlying stereotype threat effects; however, as briefly sketched above, the WM measures used so far have been unreliable and have failed to provide a precise measure of the attentional resources involved in stereotype processing. 

### 5.1. What Are Attentional Resources in Working Memory?

WM is one of the most studied topics in cognitive psychology, leading to several models differing in their conception of its functioning [77]. However, most WM models agree with the fact that attention is involved in the maintenance of information. In classic WM tasks (e.g., complex span tasks), items to be remembered must be actively maintained to resist forgetting and interference generated by distractors from the concurrent task (e.g., the processing component of the task). Information maintenance is ensured by attentional mechanisms [78,79,80,81,82]. In the time-based resource-sharing (TBRS) model [9], both processing and information maintenance rely on a common and limited attentional resource, and only one operation requiring attention can take place at a time. Therefore, when attention is occupied by processing, it is no longer available to counteract the natural decay of the memory trace, and when attention focuses on memory maintenance, concurrent processing activities must be postponed. Thus, the longer the processing of stimuli in the concurrent task is, the more attentional resources it requires, leading to an increased cognitive load of the WM task. By contrast, when processing stimuli of the concurrent task requires fewer attentional resources, i.e., each of them takes less time to be processed, the cognitive load of the task is lowered. Thus, controlling the temporal factors of the processing task is of importance, especially to investigate attentional resources in WM.

As previously mentioned, most studies in which complex span tasks were used to investigate the role of WM in stereotype threat effects had insufficient control of the processing component of the task, rendering it impossible to ensure that participants actually perform the processing task. This is problematic, since participants may have been more inclined to neglect this component of the task in order to favor its memory component. Only a few studies have reported processing task performance [15,16,83,84]. In these studies, three experiments found an effect of the threat on the processing component in young adults [16,83,84]. Only one study examined this effect in age-based stereotype threat, and did find one [83]. Furthermore, so far, no study has adjusted the processing component of the task to each individual’s WM capacity. However, it is important to do so, as, for example, stereotype threat should have no (or less) impact on low-cognitive-load WM tasks. Indeed, studies showed that low attentionally demanding tasks are not (or less) impaired by stereotype threat [39,84]. Studies conducted so far do not allow one to determine whether the stereotype threat effects can be accounted for by a reduction in attentional resources or reflect the impact of other processes. 

Nevertheless, although the WM capacity measures used to date are questionable, there is considerable evidence of a decrease in attentional resources during stereotype threat. Firstly, it has been demonstrated that low-WM-capacity individuals are more impacted by stereotype threat than high-WM-capacity individuals [26,27,28,55]. This finding suggests that stereotype threat draws attentional resources, as it affects individuals with fewer available resources more strongly. In addition, stereotype threat specifically impairs performance on high rather than low attentionally demanding tasks [39,84]. It also induces mind wandering [39,83,84,85,86,87], which are thoughts that might be attentionally demanding [14,88,89], therefore reducing WM performance [90,91]. In addition, neurophysiological studies evidenced that stereotype threat interferes with WM efficiency [92,93]. Thus, stereotype threat appears to draw on attentional resources in WM, but current measures have yet to properly assess it. 

### 5.2. Toward a Precise Assessment of Attentional Resources in Working Memory

Developing a better measure to assess attention is the first step toward better understanding the mechanisms underlying stereotype threat effects. According to the TBRS model [9], the cognitive load of a WM task represents the quantity of attentional resources required to handle the task; it corresponds to the duration of attentional capture by the task. Cognitive load depends on the time during which the processing phase captures attention, thus impeding memory maintenance. If the time duration available to maintain information during the processing phase is short, the cognitive load will be high; however, if this time duration is long, the cognitive load will be low. Thus, the ratio between the duration of attentional capture and the duration of the total task is an important factor in WM tasks. Cognitive load (CL) can be expressed as:

CL = *a*N/T, (1)

where N corresponds to the number of items to be processed; *a* to a parameter that represents the difficulty of these processes, i.e., the time during which these processes fully capture the attention; and T to the total duration of the processing task (the duration of the intermemory items interval). The TBRS model provides an easy-to-implement but very precise quantification of the cognitive load of a WM task. This measure could therefore be used to precisely quantify the impact of stereotype threat in terms of attentional resources and to determine whether or not this effect is indeed drawing resources. For example, the stereotype threat effect could be tested on low vs. high-cognitive-load WM tasks, by tailoring the cognitive load of the processing task, in terms of total number of items to be processed and single-item-processing time, to each individual’s capacity. If stereotype threat does engage attentional resources, memory or processing task performance should be more impacted when the cognitive load of the WM task is high rather than low. This would be because individuals should have fewer or no attentional resources available in the high-load condition. The cognitive load of a WM task can also be captured by measuring the time it takes to process the total number items in the processing task, i.e., the parameter *a*, when recall performance is perfect. A longer processing time reflects an increase in cognitive load. If the stereotype threat draws on attentional resources, processing it should also increase the *a* parameter.

As mentioned previously, a sticking point in stereotype threat literature is the role of WM in age-based stereotype threat effect. WM is impaired by stereotype threat when the task is depicted as measuring memory but not when the assessment is correlational. However, although some studies have not shown stereotype threat effect on WM performance, there is much indirect evidence of reduced attentional resources under age-based stereotype threat [18,19,57,76]. Thus, a more accurate and precise measure of the attentional resources involved in this effect is sorely needed. Moreover, criticism of studies that present WM measure as a memory test relies on the idea that stereotype threat impairs older adults’ performance on every task depicted as assessing memory. However, studies investigating the role of WM in age-based stereotype threat only assessed WM through recall performance [19,20], while the impact of stereotype threat on attentional resources could be captured on both the processing and memory components. 

Furthermore, this approach would provide a first step toward new insights into the different factors involved in stereotype threat. For example, emotion regulation appears to be prevalent in the decrease in WM efficiency. In addition, conclusions that self-induced suppression processes disrupt WM [14,88] were drawn from studies in which WM tasks did not properly assess nor control the cognitive load of the task. Moreover, according to Barber [22], better emotion regulation abilities with aging can explain age-related differences in mechanisms underlying stereotype threat effects. Emotion regulation would not be attentionally demanding for older adults during stereotype threat. However, its impact on WM had never been examined. Only one study has shown that older adults’ better emotional regulation ability, as assessed by questionnaires, protects them from the effect of stereotype threat on WM performance [20]. Another study challenges this hypothesis by showing that older adults no longer benefit from the positivity effect (i.e., the attentional bias of older adults toward positive information) when under stereotype threat [94]. Examining the impact of emotional regulation on attentional resources in the context of age-based stereotype threat would therefore shed new light on the underlying mechanisms. Another important debate in the literature involves the validity of the regulatory focus hypothesis to explain age-based stereotype threat [22]. Is the regulatory misfit between stereotype threat-induced prevention orientation and task orientation the underlying mechanisms of age-based stereotype threat? Several studies have shown that the effect of stereotype threat is counteracted when the task is loss-oriented [21,60,66,67,68], but the processes underlying this effect remain unknown. The findings of Worthy et al. [75] suggest a potential decrease in attentional resources caused by regulatory misfit. In this context, the use of an accurate measure of attentional resources in a situation of regulatory fit or misfit could help resolve discrepancies in the literature.

## 6. Conclusions

The phenomenon of stereotype threat is well documented, and mechanisms underlying this effect have been of particular interest. WM capacity has been examined to understand the role of attentional resources on the impact of stereotype threat on performance in complex cognitive tasks. However, the measures of WM attentional resources used to date have limitations and may not properly reflect the impact of stereotype threat on them. We propose a promising new method based on the TBRS model, to precisely investigate the role of WM attentional resources. This approach provides an opportunity toward a deeper understanding of the mechanisms underlying the effects of stereotype threat on the cognitive performance of both younger and older adults and may help to resolve the current debate in this domain.

## Data Availability

Not applicable.
